# Acceptability, Needs, Concerns, and Barriers to Digital-Based Interventions for the Prevention of Mother-to-Child Transmission of HIV: Systematic Review and Qualitative Meta-Aggregation

**DOI:** 10.2196/64816

**Published:** 2025-10-09

**Authors:** Sidik Maulana, Kusman Ibrahim, Rachel H A Arbing, Iqbal Pramukti, Annisa Dewi Nugrahani, Luh Nik Armini, Muhammad Iqhrammullah, Wei-Ti Chen

**Affiliations:** 1 Doctoral Study Program of Nursing, Faculty of Nursing, Universitas Padjadjaran, Sumedang, Indonesia; 2 Department of Medical-Surgical Nursing, Faculty of Nursing, Universitas Padjadjaran, Jl. Ir. Soekarno KM.21, Hegarmanah, Jatinangor, Sumedang, 45363, Indonesia; 3 Joe C. Wen School of Nursing, University of California Los Angeles, Los Angeles, CA, United States; 4 Department of Community Health Nursing, Faculty of Nursing, Universitas Padjadjaran, Sumedang, Indonesia; 5 Doctoral Study Program of Medicine, Faculty of Medicine, Universitas Padjadjaran, Bandung, Indonesia; 6 Department of Obstetrics and Gynecology, Faculty of Medicine, Universitas Padjadjaran, Hasan Sadikin General Hospital, Bandung, Indonesia; 7 Department of Midwifery, Faculty of Medicine, Universitas Pendidikan Ganesha, Singaraja, Indonesia; 8 Department of Chemistry, Faculty of Mathematics and Natural Sciences, Universitas Syiah Kuala, Banda Aceh, Indonesia

**Keywords:** attitude, digital health intervention, HIV, mother-to-child transmission, acceptability, systematic review, HIV transmission, patient experiences, qualitative, meta-analysis, patient needs education, support

## Abstract

**Background:**

Digital-based interventions have the potential to support initiatives for the prevention of mother-to-child HIV transmission (PMTCT). Nevertheless, reviews to explore experiences and perspectives toward digital-based interventions in mothers living with HIV remain limited.

**Objective:**

The aim of the study was to explore the experiences and perspectives toward digital-based interventions in promoting PMTCT services in mothers living with HIV.

**Methods:**

This study conducted a systematic review and qualitative meta-aggregation and adhered to the PRISMA (Preferred Reporting Items for Systematic Reviews and Meta-Analysis) and the Joanna Briggs Institute (JBI) Reviewer’s Manual. Electronic databases such as Scopus, PubMed, the Cumulative Index to Nursing and Allied Health Literature (CINAHL), and Wiley Online Library were systematically searched on July 5, 2024. The eligibility criteria included qualitative studies that focus on mothers living with HIV and health care providers, exploring their experiences and attitudes toward digital-based interventions for the PMTCT of HIV. The quality of the studies was assessed using the JBI Critical Appraisal tools for qualitative research and the Mixed Methods Appraisal Tool (MMAT) for mixed methods studies. The meta-aggregation was used to synthesize findings from included qualitative studies.

**Results:**

The 8 included studies (3 qualitative and 5 mixed-methods studies) were conducted in Kenya, South Africa, and India and evaluated mobile-based interventions such as SMS, phone calls, and mobile apps. The findings were synthesized into overarching themes: (1) positive acceptability of digital-based intervention for PMTCT services; (2) the need for integrating education, support systems, and reminders into digital-based intervention among mothers living with HIV; (3) concerns about confidentiality; and (4) personal, interpersonal, and health care–related barriers to care adherence. These themes were divided into 9 categories, including perceived satisfaction, improved adherence, the need for education, support systems, reminders, concerns about their privacy, lack of family support, financial constraints, and negative provider attitudes.

**Conclusion:**

Although most included studies were limited, their findings highlight the insight that the integration of digital-based interventions is perceived as acceptable and beneficial in strengthening PMTCT services delivery among mothers living with HIV. Mobile-based tools were valued for delivering education and reminders and facilitating communication with providers. However, concerns about confidentiality and persistent structural barriers must be addressed. To strengthen PMTCT services, it is essential to integrate user-centered digital tools into maternal care, supported by policies that ensure data privacy and equitable access.

## Introduction

Since the onset of the epidemic, an estimated 88.4 million individuals are living with HIV, and approximately 42.3 million have died from HIV-related causes [1]. By the end of 2023, around 39.9 million individuals were living with HIV globally [1]. Although the global HIV prevalence among adults aged 15‐49 years is approximately 0.8%, the burden of the epidemic varies significantly across regions and countries [1]. Pregnant and breastfeeding women, particularly in high-risk areas, remain a vulnerable group for HIV transmission [2]. HIV can be transmitted from a mother living with HIV to her child during pregnancy, childbirth, or breastfeeding [3,4]. Furthermore, pregnancies complicated by HIV infection are associated with an increased risk of miscarriage [5]. Numerous studies have shown that, without treatment, maternal HIV infection significantly raises the likelihood of stillbirth, premature birth, low birth weight, and infants being small for their gestational age [2,5-7].

Mother-to-child transmission (MTCT) of infectious diseases remains a significant global public health concern, with the prevention of HIV transmission from mother to child being one of the most pressing challenges [8]. In 2013, the World Health Organization (WHO) recommended lifelong antiretroviral therapy (ART) for all pregnant and breastfeeding women, and since 2016, also recommended its use before conception, as part of its strategy to prevent mother-to-child HIV transmission (PMTCT) [9]. To accelerate progress, the WHO Regional Office for Southeast Asia developed the “Regional Framework for Triple Elimination of MTCT in Asia and the Pacific 2018‐2030” aiming for zero new infections in children by 2030 [10]. Supporting this initiative, WHO released the third edition of its global guidelines, which detail the standards and procedures for validating the elimination of MTCT of HIV, syphilis, and hepatitis B (triple elimination). These guidelines provide uniform targets and criteria for evaluating national progress toward the elimination of one or more of these infections [10,11].

The PMTCT program is the standard of care for mothers living with HIV, which aims to enhance both maternal and infant outcomes [12]. It includes the integration of ART during pregnancy to manage HIV viremia, support retention in care, and reduce the risk of vertical transmission [13]. Continued engagement during the postpartum period is crucial for maintaining retention and achieving viral suppression, which are essential for optimal long-term health outcomes [14]. Providing ART during pregnancy and extending prophylaxis to infants during the first 6 weeks of breastfeeding has been shown to significantly reduce the risk of MTCT [15]. However, despite ongoing efforts, treatment coverage for mothers living with HIV and their children remains below the Joint United Nations Program on HIV/AIDS (UNAIDS) 95-95-95 targets for 2030 [11,16]. These gaps highlight the urgent need for effective strategies to expand access to evidence-based interventions, particularly for pregnant women, to ensure timely treatment and improve health outcomes [16].

Digital-based interventions, such as mobile health (mHealth) and telemedicine, have emerged as promising tools to support PMTCT services [17-22]. Digital-based interventions involve the use of telecommunications technologies to deliver health services remotely, encompassing a range of platforms including text messaging, websites, mobile apps, and health-related devices. Over the past decade, the integration of digital health technologies into the standard of care has grown rapidly across the continuum of HIV care [23-25]. There is growing interest in leveraging these tools to improve maternal and child health outcomes [26,27]. In low- and middle-income countries (LMIC), digital-based interventions offer innovative solutions to persistent barriers in HIV care, such as a shortage of trained health care providers, limited infrastructure, and high out-of-pocket costs for facility-based services [28]. During the HIV epidemic, digital-based interventions were widely adopted and improved provider-patient and interprovider communication, overcoming disparities in global HIV responses [29,30].

Despite the growing interest in digital-based interventions for PMTCT, evidence regarding their acceptability remains limited. Many studies have reported positive outcomes, such as increased uptake of HIV testing and adherence to ART [19,20,27,31,31-33]. Nevertheless, the feasibility and experimental aspects have often been overlooked [19,20,27,31,31-33]. A recent systematic review of 27 studies on text messaging and phone call interventions for HIV care and treatment in LMICs found showed significant favorable effects on ART adherence and increase in retention in care [34]. In addition, a meta-analysis of phone-based interventions integrating into PMTCT services showed a moderate improvement in ART [35]. These findings highlight the importance of integrating qualitative perspectives into digital-based interventions to better understand the experiences, preferences, and barriers faced by mothers living with HIV. Nurses and other health care professionals play a critical role in delivering ongoing support and personalized education through digital platforms. This approach not only fosters mothers living with HIV engagement, but also bridges the gap between technological innovation and practical, person-centered care, thereby improving maternal and their children’s health outcomes.

Understanding the feasibility of integrating digital-based interventions into PMTCT services is essential for their successful implementation. While quantitative studies provide valuable evidence on effectiveness, qualitative studies are equally important to understand the acceptability, usability, and contextual relevance of these interventions within the target population. Therefore, there is a pressing need for review studies that synthesize the experiences and perspectives of both mothers living with HIV and health care providers regarding integrating digital-based interventions into PMTCT services. This study explored the acceptability, perceived needs, and barriers related to digital-based interventions used in antenatal care (ANC) services, HIV testing, and ART adherence to support the PMTCT services. Specifically, this study aimed to: (1) identify the barriers and facilitators to the uptake and use of these interventions, (2) assess their acceptability among mothers living with HIV and health care providers, and (3) understand the treatment-related needs of mothers living with HIV.

## Methods

### Study Design

The study used a systematic review and qualitative meta-aggregation. The study adheres to the PRISMA (Preferred Report Items for Systematic Review and Meta-Analysis; see Checklist 1) and the Joanna Briggs Institute (JBI) Reviewer’s Manual [36,37]. Meta-aggregation, based on the philosophical traditions of pragmatism and Husserlian transcendental phenomenology, aims to produce generalizable recommendations to guide practitioners and policymakers [37,38].

### Eligibility Criteria

Inclusion criteria were established for this study according to the PCC (population, content, and context) framework. The population of interest consisted of mothers living with HIV and their health care providers. The content experiences and perspectives toward integrating digital-based interventions into PMTCT of HIV. Studies that reported on both positive and negative experiences and perspectives were included. Context was defined as any type of digital-based intervention (synchronous and asynchronous), such as text messaging, mobile health apps, telemedicine, or internet-based programs.

The exclusion criteria were quantitative studies of articles published in nonpeer-reviewed journals. Conference abstracts, posters, editorials, commentaries, and reviews were excluded. The articles not written in English were excluded. In addition, no date restrictions were applied to ensure the inclusion of all relevant studies regardless of their publication year. All database searches were conducted on July 5, 2024.

### Search Strategy and Study Selection

A comprehensive search of multiple databases was conducted to identify relevant studies. Scopus, PubMed, Medline, the Cumulative Index to Nursing and Allied Health Literature (CINAHL), and Wiley Online Library were searched from their inception up to 5 July 2024. The search strategy was developed using a combination of keywords and Medical Subject Headings (MeSH) terms related to digital-based interventions and MTCT of HIV. The search terms used were ([HIV OR “Human Immunodeficiency Virus” OR “Human Immunodeficiency Viruses”] AND [“Digital technology” OR “mobile health” OR mhealth OR ehealth OR telehealth OR telemedicine OR telenursing OR “mobile apps” OR “mobile application”] AND [“medication adherence” OR adherence OR “medication nonadherence” OR “medication compliance” OR “medication noncompliance”]). See Multimedia Appendix 1 for the search strategy. Additional relevant studies were identified through snowballing searches, using the “related articles” feature and the reference lists of the included articles. A total of 2 independent reviewers (SM and ADN) assessed all articles for eligibility, with any discrepancies resolved through consultation with a third reviewer (LNA). Studies were included if they contained original qualitative data on the experiences and perspectives of mothers living with HIV, health care providers, or other relevant stakeholders of digital-based interventions in PMTCT services.

### Data Extraction and Quality Appraisal

A total of 2 reviewers conducted data extraction independently using a standardized tabulation table. The table included study details such as author and year, study design, country, participant characteristics, model of digital technology, superordinate themes, subordinate themes, and quotes. Reviewers independently evaluated the studies, considering potential sources of bias, the research questions, the appropriateness of methodology, data collection, ethical considerations, rigor, data analysis, and findings. Any discrepancies between the authors were resolved through discussion and consensus.

The quality of the included studies was assessed using the JBI Critical Appraisal tools for qualitative research, and the Mixed Methods Appraisal Tool (MMAT; version 2018) for mixed methods studies [38,39]. The use of both tools was necessary due to the inclusion of qualitative and mixed methods studies. The JBI checklist is designed to evaluate the methodological validity of studies and to determine the extent to which a study has addressed the possibility of bias in its design, conduct, and analysis [38]. The MMAT is a comprehensive tool that allows for the simultaneous appraisal of qualitative, quantitative, and mixed methods studies [39]. It includes criteria tailored to assess the methodological quality of mixed methods research, such as the integration of the qualitative and quantitative components, the coherence between the research question and methodological approach, and the adequacy of the data collection and analysis methods. The authors assigned a total score to each study and divided them into three categories of quality: high (≥75%), some concerns (50%‐74%), and low (≤49%).

### Data Synthesis

Following the JBI Reviewer’s Manual, the study used a 3-step meta-aggregation approach to synthesize qualitative study findings [38]. Initially, 2 reviewers independently and repeatedly analyzed the reports of the selected studies, extracting findings and illustrative examples from the results sections. They identified themes, metaphors, and concepts described by the original authors. The credibility of the findings was then assessed and categorized as unequivocal, credible, or unsupported, based on how well the findings matched their supporting illustrations. Second, the reviewers independently grouped the unequivocal or credible findings into categories based on similar meanings. They discussed any differences and clarified the descriptions of the categories until they reached a consensus. Finally, they created synthesized findings by summarizing the categories that shared common features.

## Results

### Study Selection

**Figure 1.**
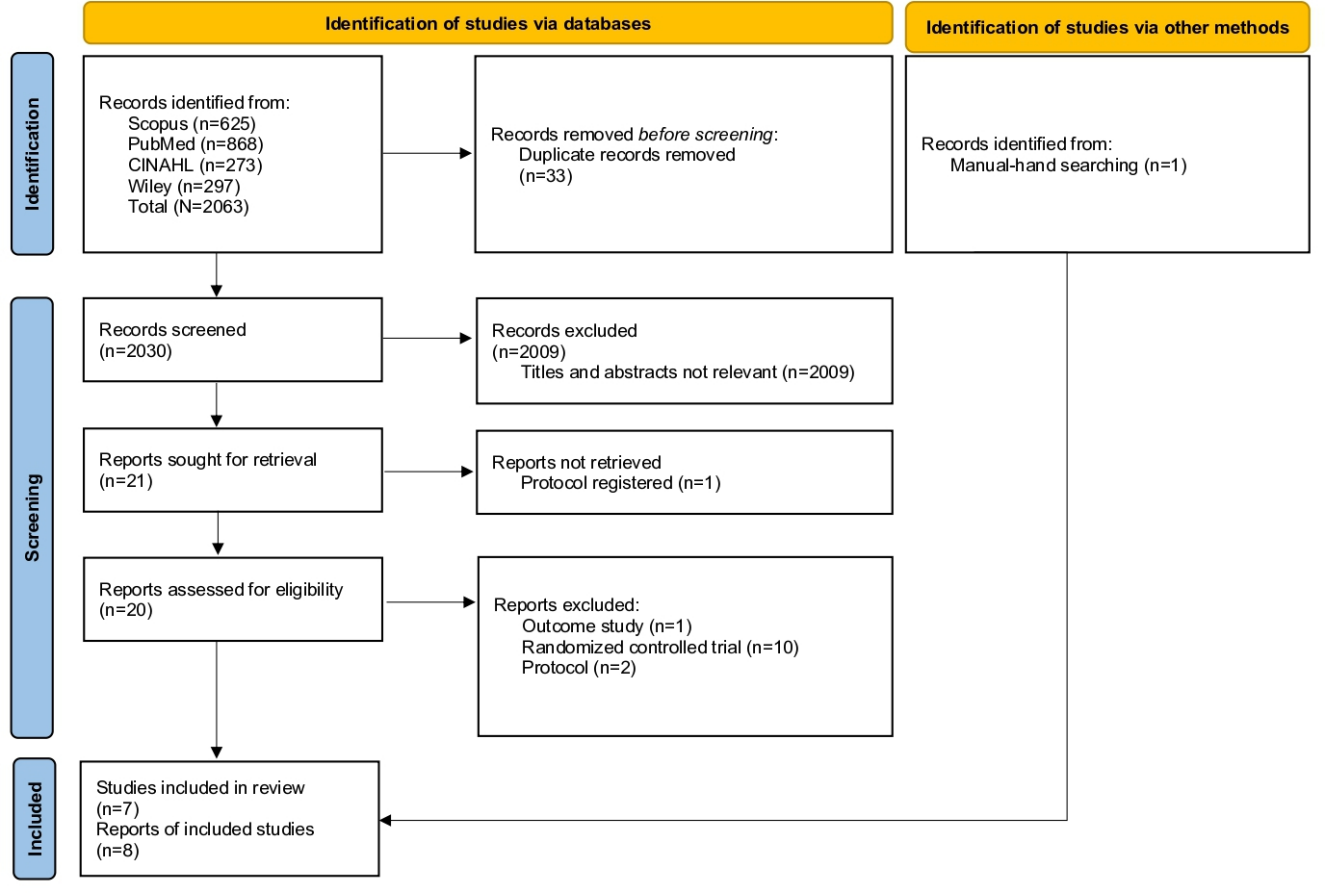
PRISMA (Preferred Reporting Items for Systematic Reviews and Meta-Analysis) flow diagram of the study selection process. Adapted from Page et al [36] (The PRISMA 2020 statement: an updated guideline for reporting systematic reviews)*.*

A total of 2063 records were identified through database searches, including Scopus (n=625), PubMed (n=868), CINAHL (n=273), and Wiley Online Library (n=297). After removing 33 duplicates, 2030 records remained for title and abstract screening. Of these, 2009 records were excluded because they did not meet the inclusion criteria (eg, not focused on PMTCT, not HIV-related, not involving digital-based interventions, or not qualitative in design). These records are reported collectively as “not relevant” in the PRISMA flow diagram, resulting in 21 reports identified for full-text retrieval. Among these 21, one study was a registered protocol or rationale paper without complete study findings and therefore excluded from the eligibility assessment. Thus, 20 full-text articles were assessed for eligibility. After full-text review, 13 studies were excluded due to reasons such as reporting outcomes only (n=1), being randomized controlled trials (n=10), and protocol studies (n=2). Consequently, 7 studies met the inclusion criteria and were included in the synthesis. An additional study was identified through snowballing, bringing the total to 8 studies included in the final synthesis. A detailed breakdown of the study selection process and exclusion reasons is presented in the PRISMA flow diagram (see Figure 1) [40-47].

### Characteristics and Quality of Included Studies

Included studies were diverse in terms of the study design, study location, and digital-based intervention model used. Among the 8 studies included, 3 were qualitative studies [41,43,44] and 5 used mixed methods [40,42,45-47]. The studies were conducted in Kenya, South Africa, and India and aligned with areas with high HIV prevalence. Digital-based interventions evaluated in these studies included mobile SMS, mobile phone calls, and mobile apps. The maternal age distribution of the study participants ranged from 18 to 32 years old. The diversity of study designs and intervention models included provided an understanding of the experiences and perspective toward digital-based interventions that addressed PMTCT of HIV (see Table 1).

**Table 1.** Characteristics of included studies.

Study	Design	Country	Number of participants	Model	Maternal age, Median (IQR^a^)	Code
Ronen et al (2018) [41]	Qualitative study	Kenya	87^b^	Mobile SMS	26	S1
Nachega et al (2016) [42]	Mixed methods	South Africa	20^b^	Mobile SMS	18‐30	S2
Van Heerden et al (2013) [40]	Mixed methods	South Africa	12^b^, 16^c^	MPAPI^d^	27	S3
Fairbanks et al (2018) [43]	Qualitative study	Kenya	87^b^, 15^c^, 30^e^	Mobile SMS	26 (23‐32)	S4
Okal et al (2022) [44]	Qualitative study	Kenya	27^b^	Mobile-phone call	N/A^f^	S5
Dean et al (2012) [46]	Mixed methods	South Africa	7^b^, 2^e^	Mobile group-SMS	18	S6
Suryavanshi et al (2020) [45]	Mixed methods	India	15^b^, 15^e^	Mobile SMS	23	S7
Simpson et al (2021) [47]	Mixed methods	Zambia	43^b^	Mobile group-SMS	22 (21‐24)	S8

a Median (IQR) reported only where the studies reported it.

b Pregnant women participants for interview or focus group discussion.

c Male partner participants for interview or focus group discussion.

d MPAPI: mobile phone-assisted personal interview.

e Health care provider participants for interview or focus group discussion.

f Not available.

Furthermore, the characteristics of the intervention model in the included studies are presented in Table 2. The interventions predominantly used mobile phone technology, including SMS-based messaging, voice calls, and mobile applications. Messaging formats ranged from unidirectional updates to interactive, bidirectional communication, enabling users to engage more actively with the system. Some interventions used group messaging platforms or private phone calls, while others relied on mobile apps specifically designed for maternal and child health support. The content of these interventions varied widely, covering areas such as appointment reminders, early infant diagnosis follow-up, counseling on ART and PMTCT, educational messages on pregnancy, breastfeeding, and disclosure, as well as support for general health and well-being. Many models also incorporated treatment adherence support and emotional or social support mechanisms. These services primarily targeted pregnant, postpartum, and breastfeeding women. They were delivered by a range of providers, including nurses, general practitioners, HIV specialists, nutritionists, trained mentors, and community health workers.

**Table 2.** Characteristics of the intervention model.

Characteristic	Number of studies, n	Reference
Intervention platform		
Mobile text–based	6	[41-43,45-47]
Mobile call phone	1	[44]
Mobile app	1	[40]
Media delivery		
Unidirectional SMS	3	[41,43,45]
Bidirectional SMS	2	[41,43]
Unspecified SMS media	1	[43]
Mobile group chat	2	[46,47]
Private phone call	1	[44]
Mobile app	1	[40]
Type of intervention delivered		
Assessment and data collection	4	[40,44-46]
Reminder (unspecified)	1	[41]
Reminder for ANC^a^ visit	1	[44]
Reminder to clients scheduled appointment	1	[44]
Reminder for EID^b^	1	[44]
ART^c^ counseling	1	[44]
Adherence counseling	1	[44]
PMTCT^d^ counseling (eg, EID)	2	[41,47]
Education (unspecified)	1	[47]
Education related to pregnancy	1	[46]
Education related to PMTCT (eg, breastfeeding, ART, and EID)	2	[45,46]
Education related to general health and well-being	1	[46]
Education related to disclosure	1	[45]
Treatment support	3	[42,44,46]
Social support	3	[41,46,47]
Target group		
Pregnant women	8	[40-47]
Postpartum and breastfeeding women	6	[40-42,44,45,47]
Target provider group		
Clinical nurse and general practitioner	3	[41,44,47]
Specialist (HIV physician, obstetrician, or gynecologist)	2	[46,47]
Nutritionist	1	[47]
Trained mentor or facilitator	2	[46,47]
CHW^e^ (includes outreach worker)	2	[44,45]

a ANC: antenatal care.

b EID: early infant diagnosis.

c ART: antiretroviral therapy.

d PMTCT: prevention of mother-to-child transmission.

e CHW: community health worker.

### Quality Appraisal

Based on the quality assessment of the included qualitative studies, a study was categorized as “Low” and another 2 were categorized as “Some concerns” (see Multimedia Appendix 2). The analysis revealed that most biases were identified in the research components related to alignment between the research methodology and the question or objectives, methods used to collect data, data analysis, and the interpretation of results.

The MMAT assessment revealed that while all studies clearly stated their research questions and collected sufficient data to address them, several methodological concerns were noted. In particular, the alignment between research questions and methodologies was often weak. The qualitative components showed unclear justification for the chosen approaches, and in the quantitative (nonrandomized and descriptive) components, issues such as limited sample representativeness, potential nonresponse bias, and inadequate statistical analyses were identified. Furthermore, the rationale for adopting a mixed methods design was frequently insufficient, with many studies failing to justify how combining methods addressed their research aims. These findings, detailed in the MMAT assessment matrix (see Multimedia Appendix 2), highlight the need for more rigorous methodological justification, especially in studies using mixed methods designs.

### Study Outcome

A total of 4 synthesized findings emerged, and 9 categories from 17 findings of this study. Table 3 shows detailed information about findings, categories, and synthesized findings. Multimedia Appendix 3 shows detailed information with original quotes.

**Table 3.** Findings, categories, and synthesized findings. S5

Finding	Category (code[s])	**Synthesized findings**
Enhances knowledge	Perceived satisfaction (S1, S2, and S4-S8)	Acceptability of digital-based intervention for PMTCT^a^ services: Mothers living with HIV have experienced positive acceptability, expressed good perceived satisfaction, and enhanced adherence (S1, S2, and S4-S7).
Enhances motivation		
Usefulness		
Easy to use		
Enhanced support system		
Helpful		
Promotes adherence	Improved adherence (S5-S7)	
Need information related to ART^b^ side effects	Need for education (S1 and S2)	Need for integrating education, support system, and reminders into digital-based intervention among mothers living with HIV: Mothers living with HIV have expressed a desire for education related to prevention and care for themselves and their baby; support system; reminders (S1, S2, S4, and S8).
Need information related to MTCT^c^		
Need information related to infant prophylaxis		
Mothers living with HIV desire support group	Support systems (S2)	
Mothers living with HIV need reminders for medication and clinical visits	Reminders (S4)	
Other people may read the message	Concern about their privacy (S1 and S2)	Concern about confidentiality: Mothers living with HIV are concerned about their privacy (S1 and S2).
Family caregiver is indifferent.	Lack of family support (S5)	Personal, interpersonal, and healthcare-related barriers to care adherence: Mothers living with HIV express that they face barriers related to family support, financial, and provider attitudes during the program (S5).
Visit to clinic when women have money	Financial constraints (S5)	
Women do not have money for meals		
Providers were displeased and even argued with mothers living with HIV	Negative provider attitudes (S5)	

a PMTCT: prevention of mother-to-child transmission.

b ART: antiretroviral therapy.

c MTCT: mother-to-child transmission.

#### Synthesis 1: Acceptability of Digital-Based Intervention for PMTCT Services

The findings of this study explored the acceptability of using digital-based interventions to deliver health information among mothers living with HIV. The findings revealed that the use of digital-based interventions was perceived as acceptable by the participants. Perceived satisfaction emerged as the central theme, with participants expressing that the information delivered through digital-based interventions was helpful and informative. The content included messages on pregnancy nutrition, the importance of breastfeeding, and reassurance about the possibility of giving birth to a healthy baby despite being HIV positive [41-47]. Another prominent theme was motivation, as participants described feeling encouraged and supported by health care providers who maintained regular phone contact [41,44]. The usefulness of mobile phones was also emphasized, with participants reporting that these tools facilitated data collection and task completion at work [40,43,44]. The ease of use of mobile phones was another major theme, with participants finding them easy to operate and flexible to interact with [40,43]. Therefore, the use of mobile phones was highly acceptable and perceived as helpful, informative, and supportive by mothers living with HIV [44].

This study yielded several findings related to care adherence among mothers living with HIV. Participants reported that counseling services remotely played a supportive role in reminding them to attend clinic appointments, adhere to medication regimens, and follow appropriate infant feeding practices [44,45]. Specifically, participants reported that counselors encouraged regular clinic visits for medication and infant weight monitoring and provided guidance on exclusive breastfeeding for the first 6 months and the importance of avoiding supplemental water [44]. Participants also reported adherence to the recommended immunization schedule [44]. These findings suggest that digital-based intervention services were both acceptable and effective in enhancing adherence to maternal and child health recommendations. In addition, the previously mentioned quotes by Suryavanshi et al [45] reported that reminder messages helped the women to remember to go get medications, demonstrating the positive impact of the counseling service on adherence to medication regimens. Another participant from Suryavanshi and colleague’s [45] study expressed their care commitment by keeping track of medication and testing schedules even without reminders [45]. These findings highlight the role of digital-based intervention in fostering a sense of responsibility and self-management among participants.

#### Synthesis 2: The Need for Integrating Education, Support System, and Reminders Into Digital-Based Intervention Among Mothers Living With HIV

This study revealed that mothers living with HIV have specific needs related to digital-based interventions, particularly in education, support systems, and reminders. In terms of education, participants expressed a desire to receive information on ART side effects [41,42], MTCT of HIV [41], infant prophylaxis [41], and the importance of medication adherence [43]. In terms of support systems, mothers living with HIV found that receiving information about peer support groups helped reduce feelings of isolation and fostered a sense of belonging [42]. In addition, digital reminders were perceived as effective tools for improving adherence to clinical appointments and medication schedules [43].

#### Synthesis 3: Concerns About Confidentiality

Concerns related to the digital integration of treatment emerged as a key theme in this study, with confidentiality identified as a prominent subtheme. Participants expressed concerns about the privacy of their HIV status and potential for stigma and discrimination if their status was disclosed [41,42]. For example, some mothers living with HIV were uncomfortable receiving SMS messages about their treatment in the presence of others who were unaware of their HIV status, fearing that this could lead to unintentional disclosure and social repercussions [41]. Similarly, some participants expressed concerns about taking ART in public places, where their status could be revealed to others unaware of their status [42].

#### Synthesis 4: Personal, Interpersonal, and Health Care-Related Barriers to Care Adherence

The findings of this study revealed that limited family support, financial constraints, and negative provider attitudes influenced access and use of HIV care integrated with digital-based interventions among mothers living with HIV. In terms of lack of family support, participants reported that having support from their family members was crucial in encouraging them to attend clinic appointments [37]. Some participants expressed how their family members would encourage them to seek treatment when they were feeling unwell. Financial barriers also emerged as a major concern, as some participants delayed treatment due to insufficient funds for transportation or medication, often relying on family members for financial assistance [37]. Furthermore, provider attitudes impacted service use, with participants reporting experiences of disrespect or poor communication from health care providers that led them to miss appointments or avoid seeking care.

## Discussion

### Principal Findings

This study identified 4 key themes that reflect the experiences and perspectives of mothers living with HIV and health care providers regarding integration of digital-based interventions into PMTCT of HIV services. First, digital interventions were perceived as acceptable, supported by subthemes such as perceived satisfaction, ease of use, usefulness, increased motivation, and improved adherence to care. Second, mothers living with HIV expressed specific needs for education, support systems, and reminders. They desired information about ART side effects, MTCT, and infant prophylaxis. Subthemes also included the importance of peer support groups and timely reminders for clinic visits and medication use. Third, concerns about confidentiality emerged as a barrier, particularly the fear of unintended HIV status disclosure through text messages received in the presence of others. Finally, participants highlighted personal, interpersonal, and health system barriers that hindered digital care adherence. These included a lack of family support, financial hardship, and negative attitudes from health care providers.

The use of digital-based interventions in health care has grown rapidly in recent years, and this study highlights their benefits in promoting adherence to maternal and child health interventions [41-47]. These interventions have increasingly become a critical component of health care delivery, even in resource-limited settings, and are now embedded within modern society [48,49]. Although some mothers living with HIV expressed concerns about confidentiality, this study found that the integration of digital-based interventions into PMTCT of HIV services enhanced their satisfaction with care and supported better adherence to treatment. This suggests the use of digital technology as an effective platform to reach and engage mothers living with HIV and their infants. As suggested previously, a high level of acceptability significantly determines successful linkage to and retention in care [50]. The findings align with the growing body of evidence on the usefulness of mobile technology in enhancing health care services across the HIV care continuum and among key populations [34,51-55].

The health care–related needs of mothers living with HIV, particularly in the context of digital-based interventions, primarily focus on education, support systems, and reminders [41-47]. This study found that mothers expressed a desire for digital-based interventions that provide educational content related to treatment, maternal health, and infant care [41]. These digital platforms can serve as valuable tools by delivering reliable information on health conditions, treatment options, medication usage, and self-care practices, thereby supporting improved health literacy and better health outcomes [56,57]. Empowering mothers with such knowledge enables them to make more informed decisions regarding their own health and that of their children.

Furthermore, mothers living with HIV, who often face complex medical and psychosocial challenges, frequently seek emotional and psychological support [58]. The theme of self-isolation frequently emerged in relation to experiences of HIV-related stigma and coping mechanisms [59]. Digital interventions that include support features, such as online communities, chat groups, or direct messaging with health care professionals can provide emotional support, reduce feelings of isolation, and foster a sense of belonging [60]. However, some participants may avoid participating in peer groups due to concerns about confidentiality.

Although mobile phone–based platforms, as digital-based interventions, were generally acceptable and facilitated care engagement, concerns specifically related to SMS were frequently reported [41,42]. These concerns were primarily driven by fears of unintended HIV status disclosure through visible text messages, particularly in shared phone settings or when notifications appeared on locked screens [41,42]. This contrast highlights that while the device itself was acceptable, the mode of digital communication, such as SMS, may introduce risks that affect trust and usability. Therefore, this is not a contradiction but rather a reflection of how acceptability is influenced by the specific design features of digital interventions.

Retention in care and adherence to treatment and care are essential for effectively managing mothers living with HIV and reducing the risk of vertical transmission through viral suppression [61,62]. In this study, mothers living with HIV frequently expressed the need for digital applications that provide timely reminders for taking and refilling medications, clinic appointments, and daily health routines. Such features can significantly enhance treatment adherence and contribute to improved health outcomes for both mothers living with HIV and their babies [55,63].

Although digital health interventions show promise in improving PMTCT outcomes, this study identified confidentiality as a major concern among mothers living with HIV [41,42]. This concern highlights the critical importance of protecting the privacy and confidentiality of health information for mothers living with HIV, especially their HIV status. This underscores the need for health care systems to protect the privacy of individuals’ health information, particularly HIV status, by implementing robust privacy policies and upholding legal and ethical standards [64]. This includes securing medical records, limiting access to sensitive data, and obtaining informed consent before disclosing health information to others, including family members or partners.

Barriers to treatment, such as lack of family support, financial difficulties, and negative provider attitudes, are critical factors influencing access to and use of digital-based HIV care interventions among mothers living with HIV. Within the context of ANC, regular clinical visits are essential; however, limited family support and financial problems often make it difficult for mothers to undergo HIV testing and access necessary health care services [65]. The cost of transportation and medical services, combined with poverty-related issues such as food insecurity, further hinders treatment adherence [66]. In addition, partner notification and effective couples counseling are essential as an entry point to identify serodiscordant partners, facilitate mutual disclosure of HIV status, and link those living with HIV to appropriate care and treatment services [67].

A strategic approach involves encouraging national HIV program leaders to advocate long-term policy changes that support the home delivery of antiretrovirals [68]. In addition, targeted financial incentives—such as unconditional cash transfers—have been shown to improve access to care and support treatment adherence, including assistance with ART initiation and transportation to health care facilities [66]. To further address economic barriers, livelihood programs and vocational training, such as microcredit initiatives, can empower mothers living with HIV [66].

In addition, provider attitudes and communication styles can significantly influence the willingness of mothers living with HIV to seek health care services. When mothers living with HIV feel judged, disrespected, or misunderstood—whether during in-person visits or digital interactions—they may be hesitant to participate fully in care or may withhold important health information. Cultural competence and empathy are essential for building trust, particularly given the unique vulnerabilities and stigma often experienced by this population [69]. Negative provider attitudes can contribute to disparities in health care access, leading to reduced quality of care and poorer health outcomes for certain patient populations [69]. To address these challenges, health care professionals should receive training in cultural sensitivity, empathetic communication, and care tailored to mothers living with HIV—both in traditional and digital settings—to ensure that integration of digital-based interventions into PMTCT services is delivered in a supportive and inclusive manner [69].

### Implications for Practices and Policies

These findings inform the design of future digital-based interventions for PMTCT of HIV by highlighting the importance of tailoring programs to the specific needs and preferences of both mothers living with HIV and health care providers. To address concerns around confidentiality, policies should ensure robust privacy protections, grounded in the ethical and legal standards of nursing and other health care professions [64]. In addition, government regulations should establish clear guidelines for evaluating the effectiveness and impact of digital health practice [70]. In addition, the national HIV program should consider implementing home delivery of antiretrovirals to address financial barriers. In addition, providing financial incentives to improve linkage to ANC and early infant diagnosis (EID), particularly among low-income populations [71,72], such as covering mobile data costs and offering transportation support for facility visits when ANC or EID is required.

Furthermore, international financial support has played a crucial role in advancing digital-based intervention for HIV care, including PMTCT initiatives. These contributions have supported progress in reducing new HIV infections and lowering maternal and infant mortality related to HIV. However, several major donor countries have announced substantial cuts to their aid budgets for the 2025‐2026 period. This financial shift may jeopardize the sustainability of PMTCT programs, particularly in low-resource settings. Strategic investment in digital infrastructure is therefore essential to maintaining momentum toward achieving global health targets, especially in remote and underserved areas. The rapid expansion of mobile and communication technologies in LMICs presents an opportunity to optimize service delivery. Digital platforms, such as eHealth and mHealth, can support timely counseling, treatment adherence, follow-up, and remote monitoring for mothers living with HIV and their infants through SMS, video consultations, or online check-ins. Strengthening these systems aligns directly with Sustainable Development Goal 3, which aims to ensure healthy lives and promote well-being for all, including ending the HIV/AIDS epidemic and improving maternal and child health outcomes.

To enhance feasibility, delivery models for PMTCT digital interventions must be tailored to local digital infrastructures and user contexts, ensuring they integrate seamlessly with existing care pathways and uphold privacy standards. In low-connectivity settings, voice calls and SMS reminders offer widely accessible options, while in higher-connectivity areas, smartphone-based tools like apps or chatbots can support more interactive counseling, peer support, and data reporting [73]. These digital platforms should be interoperable with national health information systems to monitor ANC, ART adherence, and EID outcomes. Equitable implementation also demands backup delivery modes, for instance, offering SMS as an alternative for users without smartphones.

### Limitations

Despite the valuable insights offered by this study, several limitations must be acknowledged to provide a balanced interpretation of the findings. First, methodological limitations were evident in the included studies, particularly regarding the alignment between philosophical underpinnings and research design. Many studies did not explicitly state the researcher’s cultural or theoretical positioning, nor did they reflect on how the researcher may have influenced or been influenced by the research process—an important element in qualitative rigor [74] . Second, the scope of this study was limited to 4 electronic databases (eg, Scopus, PubMed, CINAHL, and Wiley), which may have resulted in the omission of relevant studies published elsewhere, including in scientific search engines (eg, Google Scholar and ScienceDirect) and in the gray literature. This limitation may reduce the comprehensiveness and inclusivity of the synthesized evidence. However, to minimize publication and selection bias, we supplemented our search with manual hand-searching [75,76]. Third, this study included only studies published in English, which may have excluded relevant evidence from non-English sources and introduced language bias.

Fourth, in some themes, the synthesis was informed by only a single primary study, which weakens the transferability and generalizability of the findings. Conclusions drawn from a single context are less robust and may not be applicable across diverse populations or settings. Fifth, considerable variation was observed in the types of digital technologies reported across studies, which may contribute to heterogeneity in outcomes. These differences, ranging from SMS reminders to mobile health apps, can impact the consistency and reliability of the synthesized conclusions. Sixth, the search strategy did not fully use database-specific indexing terms such as PubMed MeSH headings; although MeSH terms were consulted to inform keyword development, reliance on title and abstract keyword searches may have increased the risk of missing relevant studies.

Most interventions in the included studies relied on mobile phone text (SMS) and voice-call technologies. However, 2 SMS-based studies were rated as moderate or lower in quality [41,43], which may introduce potential bias in findings related to SMS delivery models. Also, advanced digital tools were less frequently used and may present different acceptability, usability, and concern profiles compared to simpler technologies. In addition, the barriers identified regarding the integration of digital-based interventions into PMTCT services were predominantly contextual rather than technical in nature. Rather than focusing on usability or platform-specific issues, the findings revealed broader systemic challenges related to the integration of digital interventions into existing health care services. Furthermore, few studies provided technical specifications about the mobile phones used, such as device types, operating systems, or internet requirements. This lack of detail limits our ability to assess disparities in access, particularly in low-resource settings where smartphone ownership, digital literacy, and mobile network coverage are often constrained. These factors should be explored in future research to strengthen the applicability of digital-based interventions for PMTCT of HIV in diverse and underserved contexts.

### Conclusions

Findings from this study indicate that digital-based interventions, particularly mobile-based technologies, are highly acceptable among mothers living with HIV and effectively support adherence to PMTCT-related services. Mothers living with HIV valued digital tools for delivering education, emotional support, and appointments or medication reminders. However, concerns regarding confidentiality and barriers such as limited family support, financial constraints, and provider attitudes were identified as significant challenges to digital intervention uptake. To improve PMTCT outcomes in HIV, future digital strategies must prioritize confidentiality, address women’s expressed needs, and consider structural barriers.

## Supplementary material

10.2196/64816 Multimedia Appendix 1 Searching strategy.

10.2196/64816 Multimedia Appendix 2 Summary of synthesis finding, category, finding, and quotes.

10.2196/64816 Multimedia Appendix 3 Quality appraisal.

10.2196/64816 Checklist 1 PRISMA checklist.

## Abbreviations

CINAHL: Cumulative Index to Nursing and Allied Health Literature
EID: early infant diagnosis
JBI: Joanna Briggs Institute
MeSH: Medical Subject Headings
MMAT: Mixed Methods Appraisal Tool
MTCT: mother-to-child transmission
PMTCT: prevention of mother-to-child transmission
PRISMA: Preferred Reporting Items for Systematic Reviews and Meta-Analysis
