# Supplementary material for: Acceptability, Needs, Concerns, and Barriers to Digital-Based Interventions for the Prevention of Mother-to-Child Transmission of HIV: Systematic Review and Qualitative Meta-Aggregation
Source: JMIR Med Inform. 2025 Oct 9;13:e64816. doi: 10.2196/64816 (PMC12538026; doi:10.2196/64816)
Supplement: Multimedia Appendix 1 [file medinform-v13-e64816-s001.docx]

**Appendix 1.** Database search strategy

| **Database** | **Boolean search term** | **Retrieved** | **Date** |
| --- | --- | --- | --- |
| Scopus | hiv OR  "Human Immunodeficiency Virus"  OR  "Human Immunodeficiency Viruses"  AND  "Digital technology"  OR  "mobile health"  OR  mhealth  OR  ehealth  OR  telehealth  OR  telemedicine  OR  telenursing  OR  "mobile apps"  OR  "mobile application"  AND  "medication adherence"  OR  adherence  OR  "medication nonadherence"  OR  "medication compliance"  OR  "medication noncompliance" | 625 | July 5, 2024 |
| PubMed | (((((HIV)) OR (Human Immunodeficiency Virus)) OR (Human Immunodeficiency Viruses)) AND (((((((((Digital technology) OR (mobile health)) OR (mhealth)) OR (ehealth)) OR (telehealth)) OR (telemedicine)) OR (telenursing)) OR (mobile apps)) OR (mobile application))) AND ((((((medication adherence) OR (adherence)) OR (compliance)) OR (medication nonadherence)) OR (medication compliance)) OR (medication noncompliance)) | 686 | July 5, 2024 |
| CINAHL | (hiv or aids or acquired human immunodeficiency syndrome or human immunodeficiency virus ) AND ( mhealth or mobile health or m-health or mobile app or mobile application or smartphone application or app or apps ) AND ( medication adherence or medication compliance or medication non-adherence or medication non-compliance) | 273 | July 5, 2024 |
| Wiley | HIV OR Human Immunodeficiency Virus OR Human Immunodeficiency Viruses AND Digital technology OR mobile health OR mhealth OR ehealth OR telehealth OR telemedicine OR telenursing OR mobile apps OR mobile application OR web-based intervention OR internet-based intervention OR online intervention AND medication adherence OR adherence OR compliance OR medication nonadherence OR medication compliance OR medication noncompliance | 297 | July 5, 2024 |
