# Supplementary material for: Acceptability, Needs, Concerns, and Barriers to Digital-Based Interventions for the Prevention of Mother-to-Child Transmission of HIV: Systematic Review and Qualitative Meta-Aggregation
Source: JMIR Med Inform. 2025 Oct 9;13:e64816. doi: 10.2196/64816 (PMC12538026; doi:10.2196/64816)
Supplement: Multimedia Appendix 2 [file medinform-v13-e64816-s002.docx]

**Table S1.** Summary of synthesis finding, category, finding, and quotes.

| **Synthesis finding** | **Category** | **Finding** | **Quote** |
| --- | --- | --- | --- |
| Positive acceptability of digital-based intervention for PMTCT services | Perceived-satisfaction | Enhanced knowledge | Q1: “You will know how to take your medication” (Ronen et al., 2018) Q2: “I think it [SMS] will help a lot because sometimes after finding out about your status you still feel lost and don’t have any information about HIV. The only thing you know is the stuff you hear from other people so if you get SMS’s that support you it will help you.” (Nachegan et al., 2016) Q3: “What I would like to know, I thought that if you are sick then you cannot give birth to a healthy baby, but I hear that if you are on HIV care and treatment, then you can give birth to a healthy baby who is HIV negative.” (Fairbanks et al., 2018) Q4: “[Please include messages] about nutrition during pregnancy: what a woman can eat so that at the time of birth she has enough strength to deliver and enough milk to breastfeed the baby.” (Fairbanks et al., 2018) Q5: “I was told to breastfeed until 6 months. Thereafter I was to take it (baby) back for testing and checkup to introduce other meals. The nurses told me that breast milk was healthy in boosting the child immunity system for 6 months.” (Okal et al., 2022) Q6: “The baby cannot get infected because the baby takes medication and I am also taking my medication, I think there is no way…” (Okal et al., 2022) Q7: “How is it possible that the baby is going to be negative whilst you are sharing the same blood for 9 months?” (Dean et al., 2012)  Q8: “I know all the answers for your questions but first, you should watch the video so that you will get more information than me speaking to you.” (Suryavanshi et al., 2020)  Q9: “No it’s not true a pregnant woman who is HIV can give birth to a healthy baby” (Simpson et al., 2021) |
|  |  | Enhanced motivation | Q10: “We will be motivated in areas that we are weak.” (Ronen et al., 2018) Q11: “… They always check on me by calling to find out how I feed the baby and if I am having any challenges with taking drugs and if I can afford meals. They even encourage me which makes me more confident and enthusiastic…” (Okal et al., 2022) |
|  |  | Usefulness | Q12: “Using cell phones to collect data in my job would enable me to accomplish tasks more quickly...”(P1) Using cell phones to collect data would improve my job performance.” (P2) “Using cell phones to collect data in my job would increase my productivity...” (P3) “Using cell phones to collect data would enhance my effectiveness on the job...”(P4) “Using cell phones to collect data would make it easier to do my job...”(P5) “I would find cell phones useful in my job...”(P6) (Heerden et al., 2013) Q13: “I can say that [SMS] can help. My husband has refused to accept [being tested], but he helps me financially when I have hospital appointments. So I think that the SMS can help: if I give him to read he can be encouraged and he may decide to come out and know his status.” (Fairbanks et al., 2018) Q14: “I see that since we started…since I joined the program I feel it has been beneficial because when I am going through difficulties sometimes we share and talk and after sharing I feel better because I found someone that I can talk to who can give me advice….sometimes when I am sick I tell her, she tells me…she has been advising me frequently…..yes, she even tells me to go to the hospital whenever I get health complications and not to keep quiet and if I am going through any difficulties to share with her. Delayed the PNC and/or did not get the infant tested.” (Okal et al., 2022) |
|  |  | Easy to use | Q15: “Learning to operate a cell phone would be easy for me...” (P1)  “I would find it easy to get a cell phone to do what I want it to do...” (P2)  “My interaction with cell phones would be clear and understandable...” (P3)  “I would find cell phones flexible to interact with...” (P4)  “It would be easy for me to become skilful at using a cell phone...” (P5)  “I would find a cell phone easy to use...” (P6) (Heerden et al., 2013) Q16: “It is eas[ier] to share information about shameful diseases by SMS than telling the doctor face-to-face. For instance, when I have a wound in my private parts…I can send him a message and he gives me a response immediately to use a particular medicine.” Q17: “Sometimes I go to do CD4 test, and I will get the result later…so I can ask by SMS before my clinic date and get the results, so when the time for clinic reaches if the doctors asks me if I know my CD4 results I will say that it [is] like this and this.” (Fairbanks et al., 2018) |
|  |  | Enhanced support system | Q18: “If you receive the message…you will not feel lonely, you will know that there is someone who is concerned about you and that despite being HIV positive you are not alone.” (Fairbanks et al., 2018)  “created friendships amongst [themselves] where [they] would greet each other. ….They also enjoyed the face-to-face launch as they liked receiving the…, free phones and the food when we met together” (Simpson et al., 2021) |
|  |  | Helpful | Q19: “They also tell me when next I should be visiting the clinic. They even remind me on the very morning of the clinic day……..It has been helpful, as I cannot waste my time to go to the clinic when it is not the day I am supposed to visit. This also helps me note the dates and prepare adequately.” (Okal et al., 2022) |
|  | Improved adherence | Promote adherence | Q20: “I was always told (by mobile-phone Counsellor) to attend (clinic) so that I can always get my drugs to keep me strong. Anytime I came they could measure our weights and advise us how to take care of the baby.” (Okal et al., 2022) Q21: “…She (mobile-phone counselor) tells me not to be late for the clinic… (Inaudible) I should see the doctor and everything else.” (Okal et al., 2022) Q22: “She (Counsellor) told me to breastfeed until 6 months are over. I should not even give the baby water since the milk had everything the baby needed.” (Okal et al., 2022) Q23: “(I started breastfeeding) Immediately after delivery, after 3 months there were some growths that developed that could even bleed at times. They told me to stop breastfeeding and give him food. And I stopped breastfeeding after 3 months.” (Okal et al., 2021) Q24: “Yes, the baby got all of them even all the injections the baby has finished.” (Okal et al., 2022) Q25: “Yes, I did as I was advised by (name of a counselor). The first one the baby received some drops in the mouth and I took her for the rest of immunization in the clinic without delay.” (Okal et al., 2022)  Q26: “Yes... It is helpful. Like we get a reminder that if my husband is not there then at least I can go … as it had happened one time, when I was pregnant, my husband was in Pune so due to the message only, I understood that I have to go for medicines. So during that time, I went alone.” (Suryavanshi et al., 2020)  Q27: “The mobile phone is with my husband hence I am not aware of those messages but before any message, I would have remember every date testing and ART also and I do planning according to that date … ..may message be coming but my husband ignore those…maybe the message came on my old mobile also but currently that mobile is not working.” (Suryavansi et al., 2020)  Q28: “@Pavalu even me i was told that i need to buy” (Simpson, et al., 2021) |
| The need for integrating education, support system, and reminders into digital-based intervention among mothers living with HIV | The need for education | Need information related ART side effects | Q29: “They should let you know of possible side effects of certain drugs by SMS so that when you see such effects you come to the clinic immediately for help.” (Ronan et al. 2018) Q30: “If they [SMS messages] can remind us about the times of taking the pills or the dates of coming to the clinic. The SMS’s must also be educational about taking your pills and if you don’t take them, this and this will happen to you.” (Nachega et al., 2016) |
|  |  | Need information related MCTC | Q31: “I would like to know what I can do so that my child is born negative and how to take care of him so that he grows up HIV negative.” (Ronen et al., 2018) |
|  |  | Need information related infant prophylaxis | Q32: “I wish to know: the baby is tested thrice, now if it is tested and found negative will it stay negative or will the disease come back later on? That is really disturbing me.” (Ronan et al., 2018) |
|  | Support systems | Mother desire support system | Q33: “Things like [information on] support groups like this one also helping because it’s where you see that you are not alone. There are people that are going through the same problem as you.” (Nachega et al., 2016) Q34: “I think that SMS system will help more than the patient-nominated supporter; people have their own problems. What if they have a problem and they can’t make it to me? But both are good in their own different ways. And you can’t really trust people because that person can talk about you to other people.” (Nachega et al., 2016) |
|  | Reminders | Mother need to reminder for medication and clinical visits | Q35: “I think that SMS is better than the card that we are given because sometimes you leave out the card somewhere and you forget and by the time you remember it has passed by one day, and if you are reminded you will be quarreled [with] at the hospital, yet it is just forgetfulness that makes you to default.” (Fairbanks et al., 2018)  Q36: “Just like sometimes someone may forget, so you send a message to alert her that on a particular day she should be going to the clinic. So that would help her, it would also help me because I may not be at home to remind her that she should go for medicine or to the clinic.” (Fairbanks et al., 2018) |
| Concern about confidentiality | Concern about their privacy | People may read the massage | Q37: “You can send the message when I have given out my phone to someone who doesn’t know my status so if they read that message they will know my status and I think that…is not good.” (Ronan et al., 2018) Q38: “At times you go somewhere and the SMS beeps and one of your friends reads it, they might start despising you because… [they] have the old mentality that those who are HIV positive should be isolated. Some people are not free with persons who are HIV positive and that may cause stigma.” (Ronan et al., 2018) Q39: "It’s when you didn’t disclose your status and then someone get hold of your phone. I used to stay in the Eastern Cape but then I moved to Cape Town. It was easier [to take ART] because they don’t know me here. But if people don’t know about your status, it’s hard because you’re scared that people are going to see you take them.” (Nachega et al., 2016) |
| Personal, interpersonal, and healthcare-related barriers to care adherence | Lack of family support | Their family caregiver is indifferent | Q40: “Nothing, when I tell him that I am going to the clinic he tells me to just go.” (Okal et al., 2022) |
|  | Financial constraints | Visit to clinic when they have money  They do not have money for meal | Q41: “I went after five months…You know where I am going is far, so I only go when I have money, so it forced me to go after five months.” (Okal et al., 2022) Q42: “Just that sometimes when food was scarce, if I did not eat well I could see that the breast milk was not sufficient as the baby suckled after which the baby started crying…So sometimes, I called my mother, if she had money she sent it to me.” (Okal et al., 2022) |
|  | Negative provider attitudes | The providers were displeased and even argued with mothers | Q43: “…They (the providers) were not happy with me and they even quarreled with me. The following month I did not go and that is when I delivered…at home.” (Okal et al., 2022) |

References

1. Ronen K, Unger JA, Drake AL, et al. SMS messaging to improve ART adherence: perspectives of pregnant HIV-infected women in Kenya on HIV-related message content. AIDS Care. Apr 3, 2018;30(4):500-505. [doi: 10.1080/09540121.2017.1417971]
2. Nachega J, Skinner D, Jennings L, et al. Acceptability and feasibility of mHealth and community-based directly observed antiretroviral therapy to prevent mother-to-child HIV transmission in South African pregnant women under Option B+: an exploratory study. PPA. 2016;10:683. [doi: 10.2147/PPA.S100002]
3. Fairbanks J, Beima-Sofie K, Akinyi P, et al. You Will Know That Despite Being HIV Positive You Are Not Alone: Qualitative Study to Inform Content of a Text Messaging Intervention to Improve Prevention of Mother-to-Child HIV Transmission. JMIR Mhealth Uhealth. Jul 19, 2018;6(7):e10671. [doi: 10.2196/10671] [Medline: 30026177]
4. Okal JO, Sarna A, Lango D, et al. Client Experiences in a Mobile-Phone Counseling Intervention for Enhancing Access to Prevention of Mother To-Child Transmission (PMTCT) Services in Kenya. Front Glob Womens Health. 2022;3:785194. [doi: 10.3389/fgwh.2022.785194] [Medline: 35720809]
5. Dean AL, Makin JD, Kydd AS, Biriotti M, Forsyth BWC. A pilot study using interactive SMS support groups to prevent mother-to-child HIV transmission in South Africa. J Telemed Telecare. Oct 2012;18(7):399-403. [doi: 10.1258/jtt.2012.120118]
6. Suryavanshi N, Kadam A, Kanade S, et al. Acceptability and feasibility of a behavioral and mobile health intervention (COMBIND) shown to increase uptake of prevention of mother to child transmission (PMTCT) care in India. BMC Public Health. May 24, 2020;20(1):752. [doi: 10.1186/s12889-020-08706-5] [Medline: 32448299]
7. Simpson N, Kydd A, Phiri M, et al. Insaka: mobile phone support groups for adolescent pregnant women living with HIV. BMC Pregnancy Childbirth. Sep 30, 2021;21(1):663. [doi: 10.1186/s12884-021-04140-6] [Medline: 34592959]
8. van Heerden A, Norris S, Tollman S, Richter L, Rotheram-Borus MJ. Collecting maternal health information from HIV-positive pregnant women using mobile phone-assisted face-to-face interviews in Southern Africa. J Med Internet Res. Jun 10, 2013;15(6):e116. [doi: 10.2196/jmir.2207] [Medline: 23748182]
