# Supplementary material for: Acceptability, Needs, Concerns, and Barriers to Digital-Based Interventions for the Prevention of Mother-to-Child Transmission of HIV: Systematic Review and Qualitative Meta-Aggregation
Source: JMIR Med Inform. 2025 Oct 9;13:e64816. doi: 10.2196/64816 (PMC12538026; doi:10.2196/64816)
Supplement: Multimedia Appendix 3 [file medinform-v13-e64816-s003.docx]

**Quality appraisal.**

**Table S1.** Results of quality analysis of qualitative studies using the Joanna Brigs Institute (JBI) critical appraisal tool

| Component | Study | | |
| --- | --- | --- | --- |
|  | **Ronen et al. (2018)** | **Fairbanks et al. (2018)** | **Okal et al. (2022)** |
| Is there congruity between the stated philosophical perspective and the research methodology? | Unclear | Yes | Unclear |
| Is there congruity between the research methodology and the research question or objectives? | Unclear | Unclear | Unclear |
| Is there congruity between the research methodology and the methods used to collect data? | Unclear | Unclear | Unclear |
| Is there congruity between the research methodology and the representation and analysis of data? | Unclear | Unclear | Unclear |
| Is there congruity between the research methodology and the interpretation of results? | Unclear | Unclear | Unclear |
| Is there a statement locating the researcher culturally or theoretically? | Not applicable | Not applicable | Not applicable |
| Is the influence of the researcher on the research, and vice- versa, addressed? | Unclear | Yes | Yes |
| Are participants, and their voices, adequately represented? | Yes | Yes | Yes |
| Is the research ethical according to current criteria or, for recent studies, and is there evidence of ethical approval by an appropriate body? | Yes | Yes | Yes |
| Do the conclusions drawn in the research report flow from the analysis, or interpretation, of the data? | Unclear | Yes | Yes |
| Total met criteria | 3/10 | 6/10 | 5/10 |
| Quality | Low | Some concern | Some concern |

**Table S2.** Results of quality analysis of mixed methods studies using the Mixed Methods Appraisal Tool (MMAT) version 2018

| Component | Study | | | |  |
| --- | --- | --- | --- | --- | --- |
|  | **Nachega et al. (2016)** | **Dean et al. (2012)** | **Suryavanshi et al. (2020)** | **Van Heender et al. (2013)** | **Simpson et al. (2021)** |
| All type | | | | |  |
| Are there clear research questions? | Yes | Yes | Yes | Yes | Yes |
| Do the collected data allow to address the research questions? | Yes | Yes | Yes | Yes | Yes |
| Qualitative | | | | |  |
| Is the qualitative approach appropriate to answer the research question? | Unclear | Unclear | Unclear | Unclear | Unclear |
| Are the qualitative data collection methods adequate to address the research question? | Yes | Unclear | Yes | Unclear | Unclear |
| Are the findings adequately derived from the data? | Yes | Yes | Yes | Yes | Yes |
| Is the interpretation of results sufficiently substantiated by data? | Yes | Yes | Yes | Yes | Yes |
| Is there coherence between qualitative data sources, collection, analysis and interpretation? | Yes | Yes | Yes | Unclear | Yes |
| Quantitative for descriptive | | | | |  |
| Is the sampling strategy relevant to address the research question? | Yes | Yes | Yes | NA | NA |
| Is the sample representative of the target population? | Unclear | No | No | NA | NA |
| Are the measurements appropriate? | Yes | Yes | Yes | NA | NA |
| Is the risk of nonresponse bias low? | Unclear | Unclear | Unclear | NA | NA |
| Is the statistical analysis appropriate to answer the research question? | Yes | Unclear | Unclear | NA | NA |
| Quantitative for non-randomized | | | | |  |
| Are the participants representative of the target population? | NA | NA | NA | Yes | No |
| Are measurements appropriate regarding both the outcome and intervention (or exposure)? | NA | NA | NA | Unclear | Yes |
| Are there complete outcome data? | NA | NA | NA | Unclear | Yes |
| Are the confounders accounted for in the design and analysis? | NA | NA | NA | Unclear | No |
| During the study period, is the intervention administered (or exposure occurred) as intended? | NA | NA | NA | Unclear | Unclear |
| Mixed methods |  |  |  |  |  |
| Is there an adequate rationale for using a mixed methods design to address the research question? | No | No | Unclear | Unclear | Unclear |
| Are the different components of the study effectively integrated to answer the research question? | Yes | Yes | Yes | Yes | Yes |
| Are the outputs of the integration of qualitative and quantitative components adequately interpreted? | Yes | Yes | Yes | Yes | Yes |
| Are divergences and inconsistencies between quantitative and qualitative results adequately addressed? | Yes | Yes | Yes | Yes | Yes |
| Do the different components of the study adhere to the quality criteria of each tradition of the methods involved? | No | No | No | No | No |

References

1. Ronen K, Unger JA, Drake AL, et al. SMS messaging to improve ART adherence: perspectives of pregnant HIV-infected women in Kenya on HIV-related message content. AIDS Care. Apr 3, 2018;30(4):500-505. [doi: 10.1080/09540121.2017.1417971]
2. Fairbanks J, Beima-Sofie K, Akinyi P, et al. You Will Know That Despite Being HIV Positive You Are Not Alone: Qualitative Study to Inform Content of a Text Messaging Intervention to Improve Prevention of Mother-to-Child HIV Transmission. JMIR Mhealth Uhealth. Jul 19, 2018;6(7):e10671. [doi: 10.2196/10671] [Medline: 30026177]
3. Okal JO, Sarna A, Lango D, et al. Client Experiences in a Mobile-Phone Counseling Intervention for Enhancing Access to Prevention of Mother To-Child Transmission (PMTCT) Services in Kenya. Front Glob Womens Health. 2022;3:785194. [doi: 10.3389/fgwh.2022.785194] [Medline: 35720809]
4. Nachega J, Skinner D, Jennings L, et al. Acceptability and feasibility of mHealth and community-based directly observed antiretroviral therapy to prevent mother-to-child HIV transmission in South African pregnant women under Option B+: an exploratory study. PPA. 2016;10:683. [doi: 10.2147/PPA.S100002]
5. Dean AL, Makin JD, Kydd AS, Biriotti M, Forsyth BWC. A pilot study using interactive SMS support groups to prevent mother-to-child HIV transmission in South Africa. J Telemed Telecare. Oct 2012;18(7):399-403. [doi: 10.1258/jtt.2012.120118]
6. Suryavanshi N, Kadam A, Kanade S, et al. Acceptability and feasibility of a behavioral and mobile health intervention (COMBIND) shown to increase uptake of prevention of mother to child transmission (PMTCT) care in India. BMC Public Health. May 24, 2020;20(1):752. [doi: 10.1186/s12889-020-08706-5] [Medline: 32448299]
7. van Heerden A, Norris S, Tollman S, Richter L, Rotheram-Borus MJ. Collecting maternal health information from HIV-positive pregnant women using mobile phone-assisted face-to-face interviews in Southern Africa. J Med Internet Res. Jun 10, 2013;15(6):e116. [doi: 10.2196/jmir.2207] [Medline: 23748182]
8. Simpson N, Kydd A, Phiri M, et al. Insaka: mobile phone support groups for adolescent pregnant women living with HIV. BMC Pregnancy Childbirth. Sep 30, 2021;21(1):663. [doi: 10.1186/s12884-021-04140-6] [Medline: 34592959]
